# Editorial “Special Issue Clinical and Post Mortem Toxicology”

**DOI:** 10.3390/toxics12030205

**Published:** 2024-03-08

**Authors:** Eric J. F. Franssen

**Affiliations:** Department of Clinical Pharmacy, OLVG Hospital, 1091 AC Amsterdam, The Netherlands; e.j.f.franssen@olvg.nl; Tel.: +31-205999111

## 1. Introduction

This Special Issue addresses the challenges faced in detecting the exposure and intoxications of various (recreative) drugs and novel active psychoactive drugs. An increasing number of drugs have become widely available on the internet, posing the following questions: how and where can we detect these agents and their metabolites in body fluids, and, what are the potentials and limitations of comprehensive and targeted toxicology screenings in clinical and forensic toxicology cases? This issue also addresses novel options for the clinical management of intoxicated patients: prevention of (re)absorption, extracorporal enhanced elimination of drugs, and specific antidotes. In cases of post mortem toxicology, how do we interpret drug levels in blood and tissues, as well as addressing challenges related to post mortem redistribution and degradation?

## 2. An Overview of Published Articles

In their case study, Lee et al. discuss bromadiolone, a potent, long-acting anticoagulant rodenticide. Bromadiolone is often blended with cereals to produce rat bait. The study outlines an incident involving six individuals employed in a small factory who suffered from a severe bleeding tendency several weeks after consuming a rice-based meal that was tainted with bromadiolone. High serum levels of bromadiolone and excessive bleeding were found in these individuals, who were then treated with vitamin K1 in the weeks following the incident. These cases indicate that long-acting anticoagulant rodenticide may induce cumulative toxicity in repeated, low-dose exposure. Blood bromadiolone levels may indicate the need for antidote therapy (Contribution 1).

Reimerink et al. describe a case of a potentially lethal caffeine intoxication after the reported ingestion of 10 g of caffeine. Due to hemodynamic instability, due to tachycardia, hypertension, and insufficient continuous labetalol infusion, the patient was started on continuous veno-venous haemodialysis (CVVHD). After successful treatment for 15 h, CVVHD was discontinued and the patient was discharged the following day. The authors stress the importance of an early recognition of caffeine intoxication, so that haemodialysis can be considered in the case of a potentially lethal intoxication (Contribution 2).

Exhumations, conducted under legal directives, are a crucial instrument in the investigation of death allegations. In cases where the cause of death is suspected to be the result of drug misuse, pharmaceutical overdose, or pesticide poisoning, this process may be applied on human remains. However, after a large postmortem interval (PMI), determining the cause of death from an exhumed body is challenging. In this issue, Albano et al. report challenges associated with postmortem drug concentration changes following exhumation more than two years after death (Contribution 3). In their case study, they present a case of a 31-year-old man who was found dead in a prison cell. During the inspection of the scene, police officers collected two blister packs—one with a tablet and the other empty. The deceased was suspected to have taken cetirizine and food supplements consisting of carnitine–creatine tablets the evening before. No relevant autopsy findings were observed. A comprehensive toxicological analysis was performed by gas chromatography coupled with mass spectrometry. The screen results were negative for substances of abuse. However, proteomic analysis returned positive results for creatine detection and negative results for other drugs (such as clarithromycin, fenofibrate, and cetirizine). The authors present the methods, and their findings, addressing the limitations of toxicological analysis in an exhumation case with a long postmortem interval (PMI).

There has been a significant increase in sodium azide intoxications since the 1980s. Sodium azide is not regularly detected in comprehensive toxicology screenings. A specific and targeted bioanalysis is required for its detection in both blood and urine. Intoxications caused by sodium azide are becoming increasingly prevalent in the Netherlands as a result of its promotion for the purpose of self-euthanasia. The mechanism of toxicity is not completely understood, but it is dose-dependent. Van der Heijden et al. present a case of suicide by sodium azide of a young woman (26 years old) with a history of depression and previous suicide attempts (Contribution 4). The deceased was found in the presence of various prescription drugs, including temazepam, domperidone—in combination with omeprazole—and the chemical preservative sodium azide. Quantitative toxicology screening of whole blood revealed the presence of 70 µg/L temazepam (toxic range > 1000 µg/L) and 28 mg/L sodium azide (fatal range: 2.6–262 mg/L). Whole blood qualitative analysis revealed the presence of temazepam, temazepam-glucuronide, olanzapine, n-desmethylolanzapine, and acetaminophen. Interestingly, in circles promoting sodium azide, it is recommended to use sodium azide in combination with medications targeting its adverse effects, such as analgesics, anti-emetics, and anti-anxiety drugs. The medicines recovered at the deceased’s location, coupled with the results of the toxicology screens, appeared to be consistent with recommendations of self-euthanasia using sodium azide.

Synthetic cannabinoid receptor agonists (SCRAs) first appeared as a legal alternative to cannabis in 2004, and they have been linked to numerous fatalities since [1]. Simon et al. report a case of a 26-year-old male who died from consuming synthetic cannabinoid receptor agonists MDMB-4en-PINACA and 4F-ABUTINACA (Contribution 5). MDMB-4en-PINACA and 4F-ABUTINACA are potent synthetic cannabinoid receptor agonists (SCRAs). The scientific literature on the symptoms associated with these substances was evaluated, along with the pharmacological properties and possible mechanism of death. A forensic autopsy was performed according to Recommendation No. R (99)3 of the Council of Europe on medico-legal autopsies. Histological samples were stained with hematoxylin and eosin (HE). Complement component C9 immunohistochemistry was applied to all heart samples. Toxicological analyses were performed by supercritical fluid chromatography, coupled with tandem mass spectrometry (SFC-MS/MS) and headspace gas chromatography with a flame ionization detector (HS-GC-FID). The literature was reviewed to identify previously reported cases of MDMB-4en-PINACA and 4F-ABUTINACA use. Autopsy findings included brain edema, internal congestion, petechial bleeding, pleural ecchymoses, and blood fluidity. Toxicological analyses revealed the 7.2 ng/mL of MDMB-4en-PINACA and 9.1 ng/mL of 4F-ABUTINACA in the peripheral blood. The authors conclude that MDMB-4en-PINACA and 4F-ABUTINACA are strong, potentially lethal SCRA, and their exact effects and outcome are unpredictable.

The administration of intravenous lipid emulsion (ILE) is a proven antidote used to reverse local anesthetic-related systemic toxicity [2]. Although the capacity of ILE to generate blood tissue partitioning of lipophilic drugs has been demonstrated previously, a clear recommendation for its use as an antidote for other lipophilic drugs is still debated. Venlafaxine (an antidepressant which acts as a serotonin–norepinephrine reuptake inhibitor (SNRI)) and quetiapine (a second-generation atypical antipsychotic) are widely used in the treatment of psychotic disorders. Both are lipophilic drugs known to induce cardiotoxicity and central nervous depression. Cobilinschi at al. report a case of a 33-year-old man with a medical history of schizoaffective disorder, who was admitted to the emergency department (ED) after having been found unconscious due to a voluntary ingestion of 12 g of quetiapine and 4.5 g of venlafaxine (Contribution 6). An initial assessment revealed a cardiorespiratory stable patient, but the subject was unresponsive to a GCS of 4 (M2 E1 V1). In the ED, he was intubated, and gastric lavage was performed. Immediately after admission to the intensive care unit (ICU), his condition quickly deteriorated. He then developed cardiovascular collapse refractory to crystalloids and vasopressor infusion. Junctional bradycardia then occurred, followed by spontaneous conversion to sinus rhythm. Subsequently, frequent ventricular extrasystoles, as well as patterns of bigeminy, trigeminy, and even episodes of non-sustained ventricular tachycardia occurred. Additionally, generalized tonic–clonic seizures were observed, alongside supportive therapy, antiarrhythmic and anticonvulsant therapy, intravenous lipid emulsion bolus, and continuous infusion were administered. His condition progressively improved over the following hours, and 24 h later, he was tapered off the vasopressor. On day 2, the patient repeated the cardiovascular collapse and a second dose of ILE was administered. Over the next few days, the patient’s clinical condition improved, and he was successfully weaned off ventilator and vasopressor support. The authors conclude that ILE has the potential to become a form of rescue therapy in cases of severe lipophilic drug poisoning, and should be considered a viable treatment for severe cardiovascular instability that is refractory to supportive therapy. This finding is also supported by similar papers on overdoses of venlafaxine, in which blood venlafaxine concentrations were monitored resulting in enhanced clearing of venlafaxine by ILE [3].

The pharmacokinetic features of psychoactive drugs—their significant postmortem redistribution especially—challenge traditional sampling in forensic toxicology. In response to this, Sosa performed a systematic literature review to evaluate different matrices as a surrogate endpoint in the forensic toxicology of quetiapine-related deaths (Contribution 7). This review considers the results of five comprehensive studies. The highest quetiapine concentrations were usually measured in the liver tissue. As interpreted by their authors, the results of the considered studies showed a strong correlation between some matrices, but, unfortunately, the studies presented models with poor goodness-of-fit. The distribution of quetiapine in distinct body compartments and tissues showed no statistically significant relationship with the length of the postmortem interval. Furthermore, this study did not confirm the anecdotal correlation of peripheral blood concentrations with skeletal muscle concentrations. Also, there was no consistency regarding selecting an endpoint for analysis.

An alternative matrix for post mortem toxicology analysis may be fluid obtained from the chest cavity (FCC). Zughaibi et al. studied the presence of 11-nor-∆ 9 -carboxy tetrahydrocannabinol (THC-COOH) in FCC of postmortem cases collected from drug-related fatalities or criminal-related deaths to evaluate its suitability for use as a complementary specimen to blood and biological specimens in cases where no bodily fluids are available or suitable for analysis (Contribution 8). The relationships between THC-COOH concentrations in the FCC samples, age, body mass index (BMI), polydrug intoxication, manner, and cause of death were investigated. Methods: Fifteen postmortem cases of FCC were analyzed using fully validated liquid chromatography-positive-electrospray ionization tandem mass spectrometry (LC-MS/MS). Results: FCC samples were collected from 15 postmortem cases; only THC-COOH tested positive, with a median concentration of 480 ng/mL (range = 80–3010 ng/mL). THC-COOH concentrations in FCC were higher than THC-COOH concentrations in all tested specimens with the exception to bile. The median ratio FCC/blood with sodium fluoride, FCC/urine, FCC/gastric content, FCC/bile, FCC/liver, FCC/kidney, FCC/brain, FCC/stomach wall, FCC/lung, and FCC/intestine tissue were 48, 2, 0.2, 6, 4, 6, 102, 11, 5 and 10-fold, respectively. Conclusion: This is the first postmortem report of THC-COOH in the FCC using cannabinoid-related analysis. The FCC samples were liquid, easy to manipulate, and extracted using the same procedure as the blood samples. The source of THC-COOH detected in FCC could be derived from the surrounding organs due to postmortem redistribution or contamination due to postmortem changes after death. THC-COOH, which is stored in adipose tissues, could be a major source of THC-COOH found in the FCC.

Urine, vitreous humor, and bile specimens are interesting alternative matrices for post mortem toxicological analyses. Al-Asmari et al. reviewed heroin-related postmortem cases reported at the Jeddah Poison Control Center in Saudi Arabia over a 10-year period (Contribution 9). Liquid chromatography electrospray ionization tandem mass spectrometry (LC/ESI-MS/MS) was utilized to determine the 6-monoacetylmorphine (6-MAM), 6-acetylcodeine (6- AC), morphine (MOR), and codeine contents in unhydrolyzed postmortem specimens. This study assessed 97 heroin-related deaths, and they represented 2% of the total postmortem cases (median age, 38; 98% male). In the blood, urine, vitreous humor, and bile samples, the median morphine concentrations were 280 ng/mL, 1400 ng/mL, 90 ng/mL, and 2200 ng/mL, respectively, 6-MAM was detected in 60%, 100%, 99%, and 59% of the samples, respectively, and 6-AC was detected in 24%, 68%, 50%, and 30% of the samples, respectively. The highest number of deaths (33% of total cases) was observed in the 21–30 age group. In addition, 61% of cases were classified as “rapid deaths”, while 24% were classified as “delayed deaths”. The majority (76%) of deaths were accidental; 7% were from suicide; 5% were from homicide; and 11% were undetermined. The availability of urine, vitreous humor, and bile specimens provided valuable information regarding the opioids that were administered and the survival time following heroin injection.

Interpreting potential toxicological fatalities is challenging due to lack of data on drug reference concentrations in postmortem tissues. Towards this, Shaikhain et al. investigated the autopsy findings and toxicological results of fatalities involving khat in Saudi Arabia’s Jazan region from 1 January 2018 to 31 December 2021 (Contribution 10). All confirmed cathine and cathinone results in postmortem blood, urine, brain, liver, kidney, and stomach samples were recorded and analyzed. Autopsy findings and the manner and cause of death of the deceased were assessed. Saudi Arabia’s Forensic Medicine Center investigated 651 fatality cases over four years. Thirty postmortem samples were positive for khat’s active constituents, cathinone and cathine. The percentage of fatalities involving khat was 3% in 2018 and 2019 and increased from 4% in 2020 to 9% in 2021, when compared with all fatal cases. These cases all included males ranging in age from 23 to 45. Firearm injuries (10 cases), hangings (7 cases), road traffic accidents (2 cases), head injuries (2 cases), stab wounds (2 cases), poisoning (2 cases), unknown causes (2 cases), ischemic heart disease (1 case), brain tumors (1 case), and choking (1 case) were responsible for these deaths. In total, 57% of the postmortem samples tested positive for khat only, while 43% tested positive for khat with other drugs. Amphetamine was the drug most frequently involved. The average cathinone and cathine concentrations were 85 and 486 ng/mL in the blood, 69 and 682 ng/mL in the brain, 64 and 635 ng/mL in the liver, and 43 and 758 ng/mL in the kidneys, respectively. The 10th–90th percentiles of blood concentrations of cathinone and cathine were 18–218 ng/mL and 222–843 ng/mL, respectively. These findings show that 90% of fatalities involving khat had cathinone concentrations greater than 18 ng/mL and cathine concentrations greater than 222 ng/mL. According to the cause of death, homicide was the most common fatality involving khat alone (77%). More research is required, especially toxicological and autopsy findings, to determine the involvement of khat in crimes and fatalities. This study may help forensic scientists and toxicologists investigate fatalities involving khat.

## 3. Conclusions

In conclusion, this Special Issue shows the importance of comprehensive and targeted toxicology screening to detect different drugs and toxins in blood and tissues of both patients and the deceased. Quantitative measurements of drugs in body fluids are helpful in monitoring the use of antidotes and extracorporal clearance of patients in the clinic [4,5,6,7,8]. Additionally, reports on quantitative measurements of parent drug and metabolites in the body fluids of the deceased can enhance our knowledge of postmortal redistribution and ultimately aid in establishing a potential toxicological cause of death [9,10,11,12,13,14,15].
